# Cyclosporine and Extracorporeal Photopheresis are Equipotent in Treating Severe Atopic Dermatitis: A Randomized Cross-Over Study Comparing Two Efficient Treatment Modalities

**DOI:** 10.3389/fmed.2014.00033

**Published:** 2014-10-01

**Authors:** Uffe Koppelhus, Johan Poulsen, Niels Grunnet, Mette Søndergaard Deleuran, Erik Obitz

**Affiliations:** ^1^Department of Dermatology, Aarhus University Hospital, Aarhus, Denmark; ^2^Department of Clinical Immunology, Aarhus University Hospital, Aarhus, Denmark

**Keywords:** atopic dermatitis, cyclosporine, extracorporal photophoresis, comparative study, crossover study, biomarkers

## Abstract

**Background:** Severe atopic dermatitis (AD) is a recurrent and debilitating disease often requiring systemic immunosuppressive treatment. The efficacy of cyclosporine A (CsA) is well proven but potential side effects are concerning. Several reports point at extracorporeal photopheresis (ECP) as an alternative treatment modality with few and mild side effects. However, no direct comparison between CsA and ECP in the treatment of AD has been performed so far.

**Objectives:** To compare the efficacy of CsA (3 mg/kg/day) and ECP (administered two consecutive days twice a month) in a cohort of patients with severe AD.

**Methods:** A randomized cross-over study involving twenty patients with severe AD (SCORAD index 41-89) refractory to other treatments. The patients were allocated to a 4-month course of either of the two treatment modalities. Individual relapse periods (2–8 weeks) were interspersed before cross-over to the other treatment modality. Treatment efficacy was evaluated by SCORAD, PRURITUS (VAS-index 0–10), “overall global assessment” and serological biomarkers; sIL-2Rα, sE-selectin, eosinophilocytes, basophilocytes, and sIgE.

**Results:** 15 patients completed treatment. Both treatments lead to a marked and significant decrease in SCORAD and pruritus index. The average reduction of the SCORAD and pruritus index, respectively was a little higher for ECP treatment compared to CsA treatment; however, the differences did not reach statistical significance. The “overall global assessment” was significantly better in patients who underwent ECP therapy as compared to CsA treatment. None of the biomarkers showed significant changes after either treatment when compared to the initial values.

**Conclusion:** ECP administered on two consecutive days twice a month to patients with severe AD has similar potency as CsA administered daily in a moderate dose. ECP is a treatment alternative in patients with severe AD that do not tolerate or are refractory to conventional immunosuppressants.

## Introduction

Severe atopic dermatitis (AD) is a recurrent and debilitating disease often requiring systemic immunosuppressive treatment. Several immunosuppressants, including azathioprine and methotrexate, are known to exert acceptable disease control in many cases of AD. The most efficient and fast acting immunosuppressant for AD is well recognized to be cyclosporine A (CsA), which therefore often constitutes first-line immunosuppressive therapy in severe and recalcitrant cases of AD. Unfortunately, the potential side effects are concerning in relation to long-term use and efficient alternatives to CsA in AD are warranted (1–5). Extracorporeal photopheresis (ECP) is a procedure that combines leukopheresis with the administration of either oral 8-methoxypsoralen (8-MOP) prior to leukopheresis or the injection of liquid methoxypsoralen into a leukocyte-rich cell extract. In practice, the patient’s blood is removed and undergoes centrifugation in order to separate it into a fraction that is depleted of leukocytes, which is immediately returned to the patient, and a leukocyte-rich fraction. The leukocyte-rich or buffy coat fraction is then exposed to UVA light within the ECP unit and re-infused back into the patient. The entire procedure takes approximately 3 h to complete (6–8). ECP was originally introduced in the treatment of cutaneous T-cell lymphoma (CTCL) in the early 1980s. Since then, several indications for ECP have been proposed in medicine and dermatology including bullous pemphigoid (BP), connective tissue disorders, CTCL, epidermolysis bullosa acquisita (EBA), graft versus host disease (GVHD), lichen planus (chronic erosive type), multiple sclerosis (MS), pemphigus foliaceus, pemphigus vulgaris, post-transplant B-cell lymphoma, psoriasis, rejection of renal, cardiac, and lung transplants, rheumatoid arthritis, and systemic scleroderma (7). The use of ECP on a larger scale has, however, been limited to the treatment of GVDH, CTCL stage III-IV, and scleroderma (7, 9–11). In GVHD efficacy has proven comparable to combined immunosuppressant treatment with CsA and Azathioprine (12, 13). It has been suggested that the immunomodulating effect of ECP may be ascribed to restoration of Th1/Th2 imbalance thus making ECP an obvious treatment option for AD (11). The first case reports on treatment of AD with ECP was published in 1994 (14); since then several small open-labeled studies have consistently reported promising results of ECP in patients with moderate to severe AD recalcitrant to other treatments. Moreover, in these reports the side-effects appear rare and mild (6, 8, 15–18). However, controlled trials comparing ECP and CsA in AD have never been reported. In here, we report a randomized cross-over study comparing ECP and CsA as treatment modalities in moderate to severe AD. Evaluation was based on SCORAD, patient ranking of “global assessment” and pruritus. Moreover, several biomarkers earlier reported to reflect AD activity were evaluated during the trial.

## Materials and Methods

Twenty patients (age 20–45 years, 15 male, 5 female) with chronic (8–45 years) severe AD (SCORAD 40–89) were included in a cross-over trial evaluating Cyclosporine A (CsA), 3 mg/kg administered daily (Sandimmun Neoral^®^, Novartis Denmark) versus ECP (2 J/cm^2^ (Therakos UVAR^®^, Berkshire, UK) administered on two consecutive days with intervals of 14 days. The patients were initially treated with oral 8-MOP, 0.6 mg/kg, followed by photopheresis 2 h later. This regime, however, resulted in inconsistent blood levels of psoralen. Furthermore, intolerable side-effects to the oral 8-MOP were noted in our group of patients. For these reasons, clinical policy on this area was revised, and administration of 8-MOP was changed to extracorporeal administration of methoxsalen (Uvadex^®^, Therakos, Berkshire, UK) in all patients. When treated with cyclosporine, the patients underwent the following laboratory tests prior to start up and every second week during the treatment period: complete blood count, assays of electrolytes, serum creatinine (taken twice before initiation of the therapy), blood urea nitrogen, aspartate aminotransferase, alanine aminotransferase, alkaline phosphatase, γ-glutamyltransferase, lactate dehydrogenase, and bilirubin. Also the blood pressure was measured before and every second week during treatment.

Duration of treatments was 4 months each with an individual relapse period (of 2–8 weeks) interspersed. Criteria of inclusion were refractoriness to standard topical treatment (corticosteroid ointments, UVA, UVB, PUVA, tar). Criteria of exclusion were pregnancy, uncontrolled hypertension, previous malignancy, infectious disease, liver/kidney disease, or active treatment with ECP or immunosuppressants within 4 weeks prior to start of trial. Patients were randomized to either treatment after giving their written informed consent. The use of topical emollients was allowed during the course of the trial. Corticosteroid creams and ointments were allowed until 2 weeks prior to trial. During treatment, SCORAD index (19, 20) was recorded monthly. Furthermore, patients were asked to report pruritus index (on a VAS scale 0–10) during each clinical visit. At the end of each treatment course, the patients evaluated the effect on an “overall global assessment” scale (0–5) reporting their experienced grade of improvement. The following serological biomarkers were determined at the beginning and at the end of each treatment course: sIL-2Rα (pgms/mL), sE-selectin (ngms/mL), eosinophilocytes (billions/mL), basophilocytes (billions/mL), and sIgE (IE/mL). sIL-2Rα and sE-selectin were determined by means of ELISA (Abcam^®^, Cambridge, UK). The rest of the biomarkers were determined by standard analysis.

The reduction in SCORAD and pruritus index over the two treatment periods was compared between treatment modalities with correction for potential crossover effects using a linear mixed model with systematic effect of treatment sequence, treatment, and period (/carryover) and a random subject effect (PROC MIXED, SAS/STAT^®^ software, SAS Institute Inc. SAS OnlineDoc^®^ 9.1.3. Cary, NC: SAS Institute Inc, 2004).The parameters analyzed by this test are given in Table 1.

**Table 1:** **The reduction in SCORAD and pruritus index over the two treatment periods was compared between treatment modalities with correction for potential crossover effects using a linear mixed model with systematic effect of treatment sequence, treatment and period (/carryover) and a random subject effect**.

	Period 1	Period 2
Sequence CsA-ECP	Reference	+ECPxtra + carryover
Sequence ECP-CsA	+ECPxtra + sequence	+sequence + carryover

*The systematic effects were parameterized with reference in sequence group CE and first period (in which the treatment modality was CsA). Based on this reference three add-on effects (ECPxtra, sequence, carryover/period) can be estimated as illustrated in the table*.

The overall global assessment score and the results from the analysis of biomarkers were analyzed under the assumption of no crossover effects and compared between treatment modalities by, respectively, the Wilcoxon rank sum test with continuity correction and the paired *t*-test.

The trial was approved by the Danish National Committee on Health Research Ethics.

## Results

### SCORAD and pruritus

**Figure 1:**
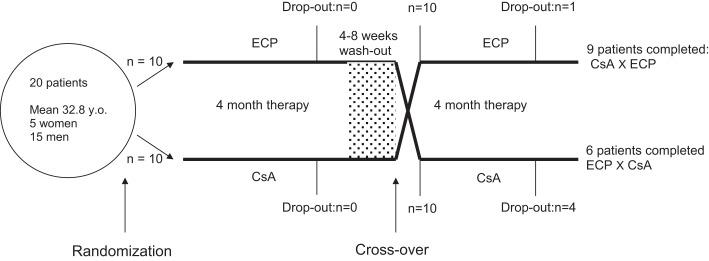
**Outline of the clinical trial set up**. For details please see the Sections “Materials and Methods” and “Results” of the text.

**Figure 2:**
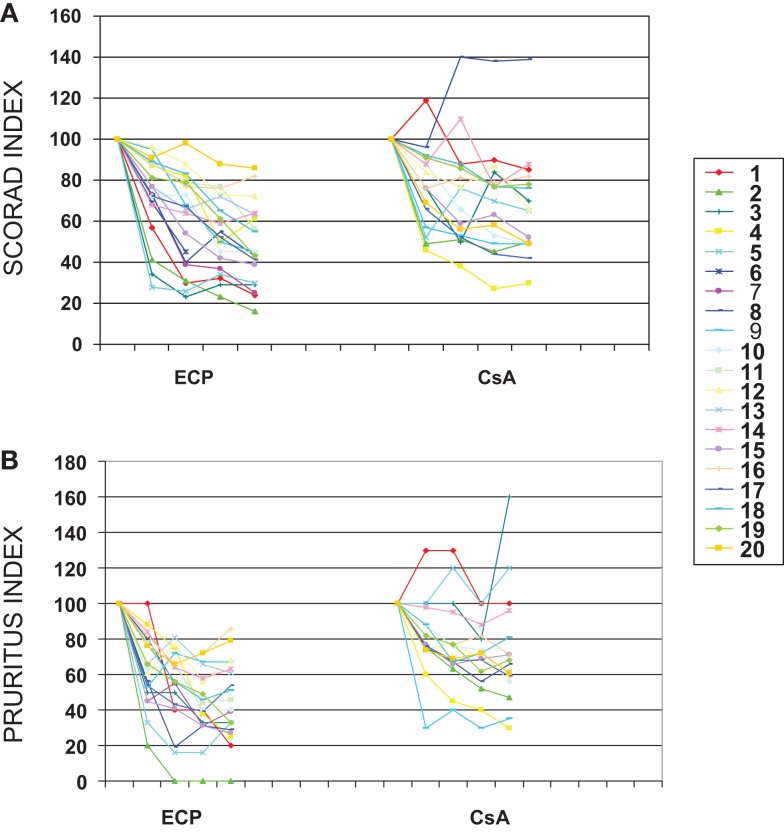
**Normalized SCORAD index (A) and normalized pruritus index (B) versus duration of treatment with either CsA or ECP**. Data from all patients completing at least 2 months of treatment with CsA (3 mg/kg/day) (*n* = 16) or ECP (2 J/cm^2^, on two consecutive days/14 days) (*n* = 20) are included.

**Figure 3:**
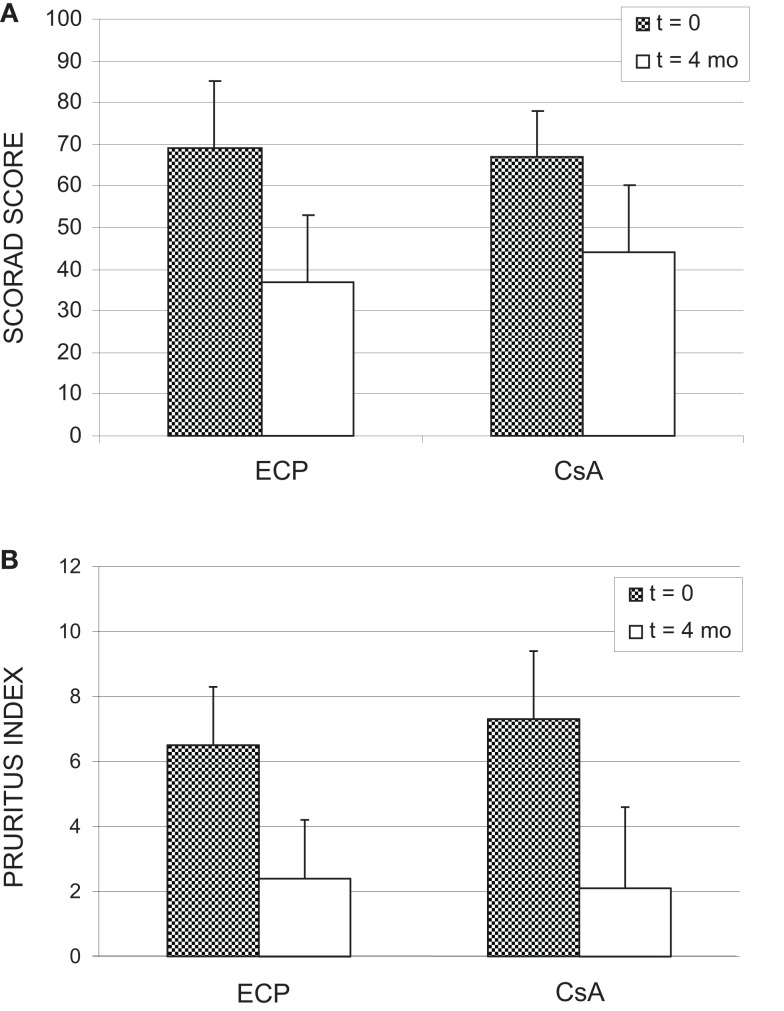
**SCORAD score (A) and pruritus index (B) before and after 4 months of treatment with either CsA, 3 mg/kg/day or ECP, 2 J/cm^2^, on two consecutive days every second week**. For details please confer the Section “Results” of the text.

The trial set-up is depicted in Figure 1. Of the 20 patients included for the trial, 15 patients completed both courses of treatment. One patient dropped out during terminal ECP treatment (due to pregnancy) and fours patients chose to leave the study during the terminal course of CsA. A number of non-severe side effects were stated as contributing factors to the decision to discontinue the treatment. However, a high degree of satisfaction with the prior ECP treatment, which often were experienced completely free of side effects, may also have added to the patients decision. All four patients who dropped out of CsA had a marked response to the prior ECP treatment (data not shown). As indicated in Figure 1, individual relapse periods of 2–8 weeks were allowed before cross-over. Relapse periods for CsA were in general found to be approximately 1–2 weeks shorter than seen for ECP (results not shown). In Figure 2, all obtained data for SCORAD (a) and pruritus (b) are shown. As can be seen, there was a clear and comparable tendency toward reduction of SCORAD and pruritus for both treatments. When similar plots were done for the separate parameters constituting the SCORAD score, similar decreasing tendencies were seen for all parameters (results not shown). Thus, a general and comparable reduction of the symptoms associated with severe AD was seen for both treatments. In Figure 3, the average SCORAD (a) and pruritus index (b) before and after each of the two treatments is shown for the 15 patients who completed both treatments. The actual numbers are given in Table 2. Substantial variation in disease severity is reflected in high variance within groups. However, a clear reduction is seen for both treatments. When considering the 15 patients who underwent both treatments, we found in ECP treated patients, one patient with a reduction of 50 SCORAD points, six patients with a reduction in SCORAD of 30–40 points, and four who showed a reduction of 20–30 points. The remainder four patients had no crucial improvement. Thus, 73% of the patients responded to ECP therapy. For CsA in this group, SCORAD diminished by 30–40 points in two patients, three patients experienced a reduction of 20–30 points, four had a reduction of SCORAD of 10–20 points, and six had no substantial reduction or even worsening of their disease. Thus, 60% responded to the CsA treatment. The reduction of SCORAD seen with ECP was marginally higher than found for CsA treatment. Among ECP treated patients, two experienced a 50 points reduction of their pruritus, one patient had a reduction of pruritus by 30–40 points, five patients experienced reductions of 20–30 points, two patients reported reductions of 10–20 points while itching was aggravated in five patients during the course of treatment. Thus, 67% responded positively to ECP. Among the CsA treated individuals, pruritus was reduced by 30–40 points in one patient, three patients had reductions of 20–30 points, five patients experienced a reduction of 10–20 points while the remainder experienced very modest improvement or even aggravation of pruritus. Thus, the positive response rate for CsA treatment was 60%. The reduction of both SCORAD and pruritus index during CsA treatment was significant (*p* = 0.0098 and 0.0013, respectively). An additional reduction in both SCORAD and pruritus was estimated with the ECP treatment [SCORAD: 6.29, CI_95%_ (−7.68; 20.2); pruritus: 0.58, CI_95%_ (−1.40; 2.56)] although statistically non-signficant in both cases (SCORAD: *p* = 0.3632; pruritus: *p* = 0.5503). None of the estimated add-on effects reached statistical significance (results not shown). Assuming no carry-over or sequence effects, the data could be analyzed by the paired *t*-test. Also this test revealed no statistical difference between the data obtained with ECP and CsA, respectively: *p* = 0.4 for ΔSCORAD and *p* = 0.5 for Δpruritus.

**Table 2:** **Patient “overall global assessment” before and after 4 months of treatment with either CsA, 3 mg/kg/day or ECP, 2 J/cm^2^, on two consecutive days every second week**.

Score	0	1	2	3	4	5	Mean ± SD
ECP (*n* = 19)	0	1	2	5	9	5	3.5 ± 1.0
CsA (*n* = 16)	1	3	5	6	1	0	2.2 ± 1.0

*Score: 0 = progression; 1 = status quo; 2 = slight improvement; 3 = moderate improvement, 4 = strong improvement, 5 = very strong improvement. For each treatment is given the number of patients having reported each of the possible scores. Moreover, the average score for each treatment are given in the table*.

### Overall global assessment

In Table 2, patients “overall global assessment” scores are summarized for all patients completing a full course of treatment of either of the two treatments. ECP was rated “good” or “very good” by 74% of participants while only 6% gave this rating to the CsA treatment. Mean score for ECP was 3.5 compared to 2.2 for CsA. This difference was statistically significant (*p* = 0.0012).

### Biomarkers

Certain biomarkers associated to AD have been claimed to be altered in response to treatment with ECP and/or CsA in AD patients. These include decrease of serum concentration of IgE and eosinophilic granulocytes in patients treated with either ECP or CsA (14, 21–23). Moreover, the serum level of sIL-2R and sE-selectin has been reported to be decreased in AD patients responding to ECP treatment (21). In an attempt to correlate these biomarkers to the efficacy of the two treatments modalities, the biomarkers were determined in each patient before and after each completed treatment modality. Basic measurement deviation and baseline values in healthy individuals were not assessed. However, the determinations of basophilic granulocytes were included as an internal marker, which is not expected to reflect AD activity. A number of samples failed thus leaving us with valid data from 10 to 12 patients in each set of data (see Table 3). As can be seen from the table, no drastic changes of any of the biomarkers were found in response to either of the treatments. When analyzed by paired *t*-test no significant differences between the results obtained with ECP and CsA was found: basophilic granulocytes: 1.21, CI_95%_: (0.74, 1.97), *p* = 0.4126; eosinophilic granulocytes: 1.10, CI_95%_: (0.63; 1.91), *p* = 0.7226; E-selectin: 0.90, CI_95%_: (0.66; 1.24), *p* = 0.4689; sIgE: 0.99, CI_95%_: (0.64; 1.53), *p* = 0.9394; sIL-2Ralpha: 1.64, CI_95%_: (0.65; 4.16), *p* = 0.2628.

**Table 3:** **Average values for SCORAD, pruritus index, sIgE, sIL-2Rα, sE-selectin, eosinophilocytes, and basophilocytes before and after 4 months of treatment before with either CsA, 3 mg/kg/day or ECP, 2 J/cm^2^, on two consecutive days every second week**.

	ECP			CsA		
	Before	After	Δ (%)	Before	After	Δ (%)
SCORAD	69 ± 16(*n* = 15; range: 50–82)	37 ± 16(*n* = 15; range: 11–66)	−46	67 ± 11(*n* = 15; range: 41–82)	44 ± 16(*n* = 15; range: 22–83)	−34
Pruritus	6.5 ± 1.8(*n* = 15; range: 5–10)	2.4 ± 1.8(*n* = 15; range: 0–6)	−63	7.3 ± 2.1(*n* = 15; range: 3–10)	4.0 ± 2.5(*n* = 15; range: 1–9)	−45
S-Il-2Rα	1650 ± 298(*n* = 12; range: 1209–4383)	1720 ± 986(*n* = 12; range: 1031–2984)	4	1756 ± 913(*n* = 12; range: 1013–4516)	1473 ± 1029(*n* = 12; range: 835–4383)	−16
S-E-selectin	74 ± 23(*n* = 10; range: 25–101)	77 ± 35(*n* = 10; range: 27–132)	4	77 ± 35(*n* = 10; range: 29–130)	76 ± 32(*n* = 10; range: 26–122)	−1
IgE	6341 ± 6509(*n* = 11; range; 103–17133)	5936 ± 6534(*n* = 11; range; 78–16.783)	−6	5742 ± 7605(*n* = 10; 353–24.459)	5224 ± 5152(*n* = 10: 155–14.166)	−9
Basophilic granulocytes	0.054 ± 0.016(*n* = 12; range: 0.03–0.09)	0.056 ± 0.014(*n* = 12; range: 0.05–0.09)	4	0.06 ± 0.015(*n* = 11; range: 0.03–0.08)	0.046 ± 0.022(*n* = 11; range: 0.00–0.08)	−23
Eosinophilic granulocytes	0.69 ± 0.39(*n* = 12; range: 0.03–1.56)	0.63 ± 0.50(*n* = 12; range: 0.14–1.9)	−9	0.85 ± 0.54(*n* = 12; range: 0.27–1.77)	0.54 ± 0.36(*n* = 12; range: 0.14–1.14)	−36

For further details please confer the text in the Section “Results.”

### Adverse effects

Extracorporeal photopheresis was well tolerated by all patients without clinically obvious adverse effects. In contrast to this, a number of adverse reactions were reported for CsA. These are summarized in Table 4. All the listed symptoms are known to be potential adverse effects to treatment with CsA. Most symptoms were relatively mild and none of the patients who redrew from the CsA treatment pointed at adverse reactions as the only reason for their drop out. No persistent increments of more than 25% were seen for any of the initial values of the laboratory test in any of the patients. Thus, we found no sign of impaired kidney function as a result of the CsA treatment.

**Table 4:** **Adverse effects to treatment with CsA registered during the trial**.

Adverse effect reported	*n*
Headaches	4
Gingival hypertrophy and/or bleeding	2
Tremor	2
Gastrointestinal symptoms	2
Flushing	2
Hypertension	1
Hypertrichosis	1
Metallic taste	2
Infections	1

## Discussion

Treatment with CsA is established as highly efficient for moderate to severe AD. Earlier reports have pointed at ECP as a valid alternative. In this study, we have compared standardized courses of ECP and CsA, respectively. The obtained results allow us to conclude that both treatments are efficacious for severe AD. Moreover, the results show that under the given conditions, ECP was equipotent to the applied CsA regime in reducing disease activity as measured by SCORAD and pruritus index, respectively. In fact, a tendency of ECP being marginally more potent than CsA may be traced from the data. However, certain circumstances may have contributed to a seemingly lesser effect of CsA compared to ECP. Serum cyclosporine values or metabolism was not examined. Such an analysis would have helped assessing effect and compliance with respect to the CsA treatment. Also, CsA dose needed to treat AD may vary depending on patient susceptibility as well as the period of time of the treatment. Former clinical trials have thus used CsA in higher doses (1, 4, 24–26). In this trial, we used the recommended starting dose. ECP was administered with an optimized frequency and dosing (15). Moreover, although not significant in our statistical analysis, minor carry-over effects cannot be excluded. Thus, a positive carry-over effect from the CsA treatment modality to the ECP treatment modality in the CsA-ECP sequence group could not be excluded.

The drop out of CsA treatment amounted to 40% among the patients who had completed ECP therapy as their first course of treatment. This was probably in part due to greater satisfaction with the prior ECP treatment. The high satisfaction with ECP was also reflected in significantly better “patient overall global assessment” score for this treatment. Compliance to ECP therapy was remarkably high. Adherence to this treatment may have been ameliorated by the fact that treatment is “tablet-free.” Moreover, the high compliance to ECP treatment being the more time consuming may also have been improved by the fact that many patients were out of job due to their extensive AD disease.

The pathogenesis of AD is complex and involves genetically determined epidermal barrier dysfunction and characteristic immunological deviations. The latter include elevated serum IgE levels, eosinophilia, and T-helper type 2 (Th2)-skewed cytokine patterns in the acute phase of the disease and increased numbers of circulating regulatory T cells. sIL-2Rα and sE-selectin have been claimed to correlate to disease severity (SCORAD) and to efficacy of treatment with CsA and ECP in previous studies (21, 27). Also, a decrease in IgE and eosinophilic granulocytes have been claimed in response to these treatments (22, 23, 28–30). Our results did not confirm these findings. In fact, only small and insignificant changes were found for all the tested biovariables. The more prominent (but statistically insignificant) decrease in serum concentration of eosinophilic and basophilic granulocytes in CsA treated patients may simply represent a more or less generalized inhibitory effect of CsA on lymphocyte proliferation and not a cellular reflection of disease activity at a given time. However, taken together our results lacked statistical strength and more data correlating serological markers, disease activity of AD, and perhaps histology in patients treated with ECP and CsA, respectively are warranted.

In summary, we found a standard treatment with ECP to exhibit similar potency to CsA therapy given in a dose of 3 mg/kg/day for moderate to severe AD. The profile of safety for long-term treatment with ECP is only reported in few small studies (6) but side effects are seemingly rare and mild and B- and T-cell immunity is left unaltered (18, 31). Nevertheless, due to the cost being five times higher for ECP treatment than treatment with CsA, immunosuppressants will probably remain the mainstay in patients needing lengthy treatment of AD. However, ECP may serve as a valuable alternative to systemic treatments with immunosuppressants when such treatment is not tolerated (e.g., due to side effects) or acceptable for the patient.

## Conflict of Interest Statement

The authors declare that the research was conducted in the absence of any commercial or financial relationships that could be construed as a potential conflict of interest.
